# Communication and Therapy Planning for Patients of Reproductive Age Under Immunomodulatory Treatments for Psoriasis or Psoriatic Arthritis—Survey of the German National Psoriasis Registry PsoBest

**DOI:** 10.3390/healthcare13091017

**Published:** 2025-04-28

**Authors:** Brigitte Stephan, Christina Sorbe, Birgit-Christiane Zyriax, Janne Schmittinger, Matthias Augustin, Rachel Sommer, Neuza Maria Bernardino da Silva Burger, Ansgar Weyergraf, Ralph von Kiedrowski, Laura Kühl

**Affiliations:** 1 Institute for Health Services Research in Dermatology and Nursing (IVDP), University Medical Center Hamburg-Eppendorf (UKE), 20251 Hamburg, Germany; 2 Midwifery Science—Health Services Research and Prevention, Institute for Health Services Research in Dermatology and Nursing (IVDP), University Medical Center Hamburg-Eppendorf (UKE), 20251 Hamburg, Germany; 3 Outpatient and Studycenter on the Hase Gbr, 49565 Bramsche, Germany; 4 Company for Medical Study & Service Selters GmbH, 56242 Selters (Westerwald), Germany

**Keywords:** pregnancy, biologics, systemic therapy, skin disease, dermatology

## Abstract

**Background/Objective:** During the systemic treatment of moderate to severe psoriasis in patients of reproductive age, contraindications and therapeutic peculiarities must be taken into account. Doctor–patient communication is crucial for therapy conduct and compliance. **Methods:** This survey among male and female patients from the German psoriasis registry, PsoBest, aims to provide real-world evidence on communication and patient needs for those of reproductive age (18–55). **Result:** In total, 404 patients were eligible for the analysis (254 m, 150 f), including 39 patients currently wishing to conceive (20 m, 19 f). Patients with and without the desire to have children received similar systemic therapy. In most cases, treatment was not adapted when patients expressed a desire to have children (85.9% m, 79.5% f). Only 38.3% of men and 49.9% of women had been informed about options for conception during or before systemic therapy, mainly by dermatologists (77.4% m, 84.6% f). The majority of patients retrieved additional information about the wish to conceive and medications from the internet and other media. This survey emphasizes the importance of open communication between patients and physicians regarding family planning and therapy options. **Conclusions:** Physicians need to broach the topic of family planning, conception and pregnancy with patients and support with evidence-based information to enable comprehensive medical decision making and safe therapy choices.

## 1. Introduction

Our knowledge about psoriasis as a chronic inflammatory disease has changed dramatically during the last two decades; the disease is no longer seen as limited to the skin but involves immunological mechanisms that impact several organ systems [1,2]. Therefore, modern therapies aim to target the inflammation using systemic medications, with providers tending to prescribe these interventions earlier in the disease trajectory [3,4,5,6,7].

About half of the patients with psoriasis or psoriatic arthritis are under the age of 55 and possibly concerned with family planning and the interference of the disease activity with their social life or family concept. With effective treatment and modern systemic therapeutics, the goal of clear skin and lack of inflammatory activity has become a real possibility, even for patients with severe disease.

Within the last 10 years, our arsenal of systemic medications approved for psoriasis has expanded rapidly, and we have anti-inflammatory medications, which can be adapted to individual patient-centred needs [8,9,10]. Approvals for systemic medications include information about compatibility with conception, pregnancy and breastfeeding and differ significantly between therapeutics. Therefore, treating physicians should address this topic to patients of potential age for family planning. The choice therapeutic plan in this vulnerable life phase should consider all options and alternative treatment regimens to weigh the benefits from adequate anti-inflammatory control against the risks of therapeutic medications. From safety data as well as registries, we gained evidence-based knowledge about special therapeutics, and it is mandatory to use this information to guide the proper choice of medications.

Information for patients is heterogeneous, deriving from experiences, non-scientific sources, online research and social media. Physicians are not always aware of this competing situation. To date, there is no prior study addressing how patients with psoriasis and family planning goals seek information from additional sources instead of discussing the topic with their physician.

The German National Psoriasis Registry PsoBest includes patients with moderate or severe psoriasis and/or psoriatic arthritis at the initiation of systemic therapy and follows them for up to 10 years, regardless of the further course of treatment. Data are collected via standardised questionnaires in dermatological practices and clinics as well as via mailed patient interviews. The up-to-date registry follows more than 24,500 patients nationwide. The registry regularly starts surveys for active patients with follow-up visits focusing on different research questions, e.g., regarding disease, therapy, safety concerns or patient needs. To date, no studies of needs regarding information and therapy modifications for patients with psoriasis or psoriatic arthritis and the wish to conceive have been conducted. The present study focused on special needs regarding information about therapy options and modifications for patients with psoriasis or psoriatic arthritis and the wish for family planning. The analyses are intended to gain insight in ideas, plans or experiences with communication and information about family planning in the context of the psoriasis disease.

## 2. Materials and Methods

This analysis was based on patients registered by 31 December 2023 in the PsoBest database with quality-assured and validated data. Men and women of reproductive age (18 to 55 years) were selected. Regarding the probability of spontaneous conception, women are more likely to experience a pregnancy under the age of 45 [11]. To account for fertilisation management in female patients as well as a longer duration of fertility in male patients, the age threshold was set to 55 years at maximum.

There was no further pre-selection or controls, and the questionnaires were gender-specific (e.g., female or male versions; see Supplementary Material). It was a cross-sectional, descriptive analysis of a set of 10 questions. The questionnaire was pilot tested for comprehensibility and manageability in clinical routine care in a small patient group (3 men and 2 women). After a positive vote from the Ethics Committee of the University Hospital Hamburg-Eppendorf, the survey was sent out together with routine PsoBest questionnaires between October 2023 and March 2024. At least 1000 people were sent questionnaires. There was no a priori calculation of sample size, since this was an exploratory analysis.

The statistical description of each questionnaire item was based on standard statistical measures. For categorical data, absolute and relative frequencies (valid percent) were computed. The number of missing values and, thus, the number of valid responses differed from item to item. Percentages and numbers were given for each relative frequency. For metric data, minimum, maximum, mean and standard deviation (SD) were computed. The homogeneity between the groups of responders and non-responders to the survey was examined with independent sample t-tests (for metric data) and chi-squared test (for categorical data) with a *p*-value alpha = 0.05 as the level of significance. Fisher’s exact test was used to analyse the differences between patients with and without the desire to conceive. To account for multiple testing, *p*-values were adjusted following the Bonferroni correction. Missing data were not imputed. The analysis was performed with IBM SPSS Statistics Version 29 (IBM Corp., Armonk, NY, USA).

## 3. Results

**Figure 1:**
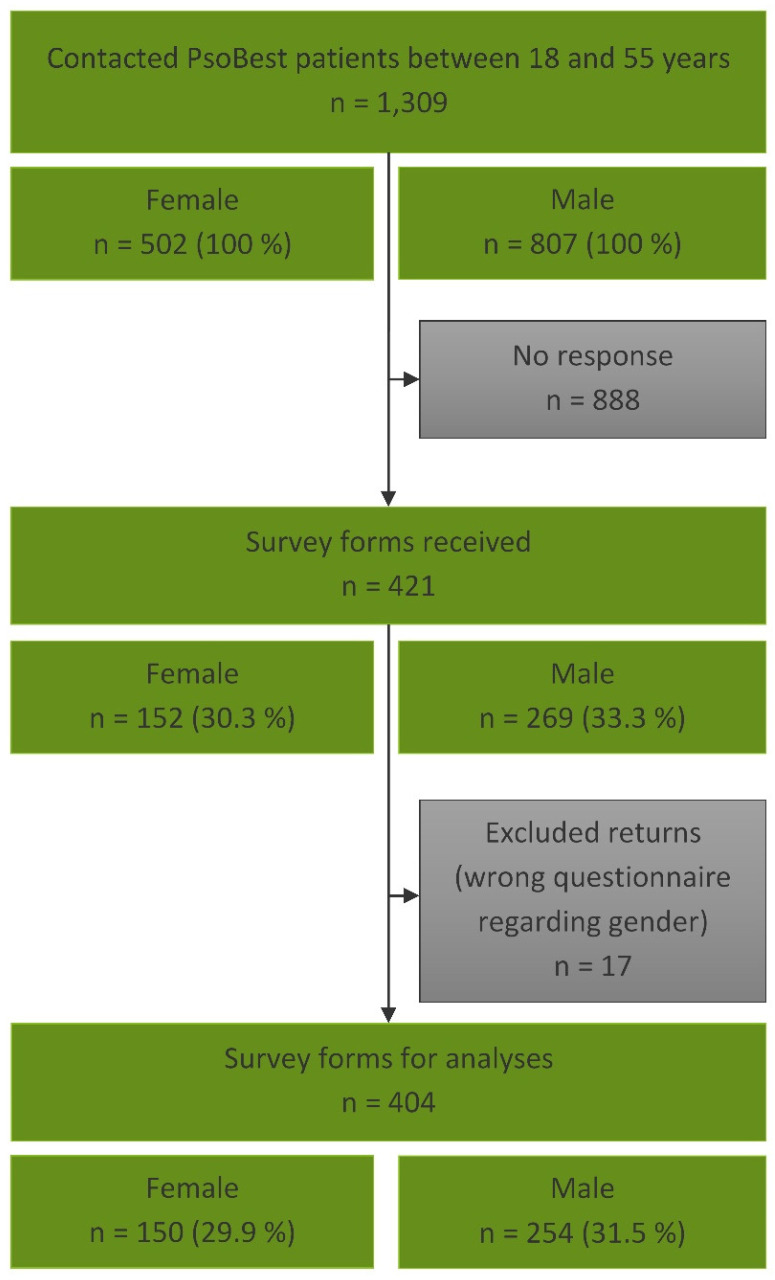
Sample size collection until 31 March 2024. Out of *n* = 1309 patients contacted, data from *n* = 404 were eligible for analysis.

The survey started in October 2023 and we contacted 807 male and 502 female patients consecutively through March 2024. After 269 men and 152 women had responded (response rate 33.3% and 30.3%, see Figure 1), we discontinued the distribution of questionnaires. There were 17 questionnaires excluded from analysis because the patient filled out the wrong gender questionnaire. The final sample used in analyses included 254 males (31.5%) and 150 females (29.9%).

The baseline parameters at PsoBest visit 1 were compared between survey responders and non-responders separated by gender to assess the possible selection bias (Table 1). The groups only differed slightly, which indicates a low selection bias within the entire PsoBest cohort. The responders were slightly but significantly older: the mean age for male survey responders was 38.4 years (±7.4) vs. 36.9 years (±8.3) for survey non-responders (*p* ≤ 0.014). The mean age for female survey responders was 37.6 years (±7.7) vs. 35.8 years (±8.4) for survey non-responders (*p* ≤ 0.026). The severity of disease at entry into the registry measured as the proportion of patients with psoriatic arthritis (PsA), Psoriasis Area and Severity Index (PASI) and Body Surface Area (BSA) did not differ significantly between survey responders and non-responders. The male survey non-responders had a higher burden of disease than responders. The Dermatology Life Quality Index (DLQI) amounted to 12.1 (±7.6) for male survey non-responders vs. 10.5 (±6.8) for responders (*p* ≤ 0.005). There were no significant differences in DLQI among women.

### 3.1. Reproductive Intentions in Patients with Psoriasis or Psoriatic Arthritis Under Immunomodulatory Treatment

Most of the patients under systemic therapy did not have a current wish to conceive. Of all responders to the survey, 8.0% of men (*n* = 20) and 13.2% of women (*n* = 19) reported a desire to conceive. The majority stated that they intended a pregnancy spontaneously (men: 68.4%, *n* = 13; women: 56.2%, *n* = 9). Almost all patients received current systemic therapy against psoriasis (men: 96.0%, *n* = 239; women: 83.6%, *n* = 112). When patients indicated a desire for family planning, they had a broad and similar range of medications than those who did not have a desire for family planning (Table 2). Patients who wish to conceive are prescribed biological therapy slightly more often than their counterparts (men: 85.0% (*n* = 17) vs. 82.9% (*n* = 190), *p* > 0.999; women: 68.4% (*n* = 13) vs. 64.8% (*n* = 81), *p* > 0.999). Non-biologic treatment was observed in 10.0% (*n* = 2) and 8.7% (*n* = 20) of men with and without a current wish for conception, respectively, and in 5.3% (*n* = 1) and 12.0% (*n* = 15) of women (both *p* > 0.999).

A large proportion of patients (men: 85.9%, *n* = 171; women: 79.5%, *n* = 70) did not report a change in therapy when they expressed their wish to conceive. In total, 2.0% of men (*n* = 4) and 11.4% of women (*n* = 10) reported that their medication was discontinued before conceiving, and 0.5% of men (*n* = 1) and 1.1% of women (*n* = 1) reported that another medication was chosen.

Overall, a low incidence of pregnancies was reported: 13.4% of men (*n* = 33) and 7.1% of women (*n* = 9) declared conceiving under systemic therapy. The total number of fathering under immunomodulatory treatments was 47 (in 254 men), and the total number of pregnancies was 20 (in 100 women). More biologic therapies were prescribed during fathering respective conception than non-biologic therapies (Table 3). Men most frequently received Secukinumab (*n* = 9) and Adalimumab (*n* = 9) at the time of fathering. Women most frequently received no therapy at the time of pregnancy (*n* = 7). If a medication was taken, it was most often Certolizumab (*n* = 3).

In comparison to all patients registered in PsoBest between 2018 and 2019 (data collection prior to COVID-19 pandemic), male survey participants with an event of fathering were prescribed biologics more frequently (65.9% vs. 48.2%). However, women with an incidence of pregnancy less frequently received a biological therapy than the comparative group from PsoBest (40.0% vs. 44.9%). Certolizumab was seen more often in the survey than in the total registry data (15.0% vs. 2.9%). Statistical comparison between survey and total registry data was not performed, since patients may be part of both data sources and might have experienced more than one event.

### 3.2. Patient Counselling on Reproductive Health During Immunomodulatory Treatment

In 61.7% of the men (*n* = 150) and 51.1% of the women (*n* = 68) surveyed, the physician in charge did not address the issue of conception during immunomodulatory treatments. Consequently, only 38.3% of men (*n* = 93) and 49.9% of women (*n* = 65) had been informed about conception during or before therapy start. The gender of the attending physician did not seem to have any influence on this: among physicians who initiated the discussion about family planning, 51.6% (*n* = 79) were male and 48.4% (*n* = 74) female.

**Figure 2:**
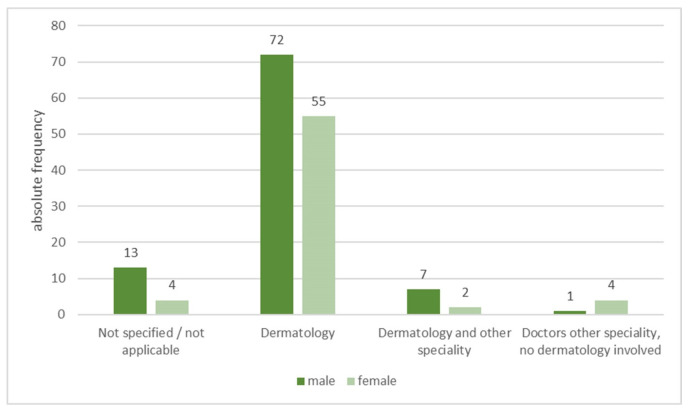
Specialists informing for family planning possibilities. According to the patients surveyed who suffer from psoriasis, dermatologists were the most frequently named specialists.

If the topic had been addressed, it was mainly by dermatologists (see Figure 2). Also, 77.4% of men (*n* = 72) and 84.6% of women (*n* = 55) were informed by their treating dermatologists. Other respondents mentioned a combination of dermatologists and other treating physicians like gynaecologists and rheumatologists (men: 7.5%, *n* = 7; women: 3.1%, *n* = 2).

According to free-text information from patients, doctors addressed the general clarification of the desire to conceive or informed the patient that the therapy is contraindicated in case of conception and a change to another therapy would be necessary.

The majority of patients (men: 60.3%, *n* = 140; women: 63.2%, *n* = 72) felt well informed by their doctors about their psoriasis treatment and the wish to conceive. In the subgroup of patients with a current wish to conceive, 55.0% of men (*n* = 11) and 63.2% of women (*n* = 12) felt well informed.

Almost all patients obtained further information regarding fathering, conception or pregnancy (men: 94.6%, *n* = 227; women 94.4%, *n* = 119). Most of them did not provide a source (men: 94.3%, *n* = 214; women 94.1%, *n* = 112), but if they did, the internet was the most frequent source of information. Moreover, the pharmaceutical manufacturer Embryotox, other doctors, or a combination of different sources were named.

The number of reported pregnancies were limited, and we cannot detect special risk signals like congenital malformations or preterm birth. The spectrum of reported adverse events reflects the pregnancy outcomes for patients with psoriasis disease [12,13,14]. The majority of women had no complaints during the course of pregnancy (*n* = 10). Seven women suffered from general complaints, and two women had severe complaints that required hospitalization. Complaints that were mentioned were vomiting (*n* = 7), nausea (*n* = 4), high blood pressure (*n* = 2) and premature labour (*n* = 2). A herniated disc was reported once.

### 3.3. Perceived Disease Severity in Women During Pregnancy

Pregnancy was more often associated with a worsening of psoriasis or psoriatic arthritis severity or symptoms than an improvement. During the first trimester, seven women indicated worsening of the disease, five reported no changes, and only one woman experienced an improvement in disease during pregnancy. After delivery, there was a trend of unchanged disease activity (*n* = 7), one woman reported an improvement in disease and four women experienced greater disease activity.

### 3.4. Perceived Impact of Psoriasis on Conception

The majority of patients who became parents (men: 60.6%, *n* = 20; women: 55.6%, *n* = 5) felt that their skin disease had no influence on fathering or pregnancy. A small number (men: 6.1%, *n* = 2; women *n* = 0) were not sure about the influence of the disease. A majority of patients (men: 69.0%, *n* = 20; women: 75.0%, *n* = 6) did not talk to their doctor about it.

## 4. Discussion

The present study aimed to recruit female and male patients with psoriasis equally. There was no significant difference in sociodemographic data between responders and non-responders to the survey. Female and male respondents were comparable in mean age and in severity of disease by PASI, BSA and DLQI, so we concluded that there was no bias from these data in responding and showing, therefore, interest for this topic answering questions about family planning in the presence of psoriasis with the need for systemic therapy. The scores for severity reflected moderate to severe disease in all groups, which was expected for baseline data at entry into the registry. From this, we anticipated a significant impact on life and psychosocial impact, as we know from other studies, which is relevant for the question of planning life and family [15,16,17,18,19].

The majority of responders to the survey did not declare a wish for conceiving under systemic therapy (question 1). However, 8% of male (*n* = 20) and 13.2% of the female responders (*n* = 19) did report a desire for conceiving, despite their burden of inflammation. Chronic inflammatory diseases are found to have an impact on conception, pregnancy conduct and outcomes [12,13,14,20,21], and patients should be cautiously informed about this circumstance. This does not only apply for skin diseases and is also well known for other chronic inflammatory disease like inflammatory bowel syndrome, rheumatoid arthritis or thyroid autoimmunity [22,23,24,25,26,27,28]. Physicians care for patients under systemic therapies, and in the case of an unfavourable event in the course of a pregnancy, patients should be aware of the possibility that a negative event may occur due to the disease itself, not necessarily due to the therapy. This topic can be highly sensitive, making it difficult for patients to discuss, especially if they are struggling to conceive and work in collaboration with fertility clinics. Conception and pregnancy planning can be worrisome and create a tense situation. In reflecting this relevant need in this survey, physicians should address this actively in their visits.

Women were not more likely to address family planning in visits than men, which underlines observations about common uncertainty in fertility intentions in more female than male patients [29]. This study demonstrated that patients who indicated their desire to conceive, men and women patients alike, did not feel that this impacted their choice of therapy (question 8). For patients who reported undergoing systemic medication, the choice of medication did not significantly differ between groups with or without a wish for family planning. Male as well as female patients received a comparable spectrum of systemic treatments. There was a tendency for a higher percentage of patients treated with TNF alpha inhibitors in the group of patients with a current wish to conceive, which, by guidelines, are recommended, preferably before other biologics in the case of intended conception [6,7], but we also found a number of patients receiving methotrexate, which is contraindicated in pregnancy and should be discontinued early before conception. This might signal that conception and family planning topics were not discussed with the patient before initiating systemic medication.

Although only a small number of our cohort reported pregnancies while receiving systemic therapy, we have their descriptive statements about the subjective influence of treatment on conceiving and pregnancy. With regard to the few pregnancy outcomes reported in this survey (*n* = 20), we cannot identify special risk signals.

The results revealed that respondents who received professional information from a physician regarding therapeutic options that coincide with family planning mostly received this information from their dermatologist and less frequently a physician from other disciplines. Patients did not necessarily address the wish to conceive, although they felt an influence of their psoriasis on conceiving or the severity of disease during pregnancy (question 7). This emphasizes the need for dermatologists to actively address the wish to conceive and therapy options during visits. Moreover, the survey revealed that the majority of patients retrieved additional information about the wish to conceive and medications from the internet and media, rather than relying on their physician. The quality of these sources can be questionable, and prior evidence suggests an inverse correlation between content quality and number of viewers (clicks) [30,31,32,33]. This can lead to misunderstandings or unnecessary or wrong therapy decisions that are misaligned with family planning goals.

Despite high scores indicating inflammatory activity, we found a number of male patients (2%, *n* = 4) who stopped medications before conception without an urgent need to do so. Prematurely ending therapy can have short- and long-term therapy implications, reducing control over the disease and its inflammatory activity. Treatment plans should aim to avoid unnecessary interruptions due to possible negative impacts on the course of the inflammation. In women, a high burden of inflammation during pregnancy poses as risk to mother and child, which has to be kept in mind [34,35,36]. Physicians’ uncertainty about treatment options should not lead to neglecting proper therapy with increased inflammation and consecutive complication of a well conducted pregnancy and delivery.

Biologics for the treatment of psoriasis are not found to pose any risk signals for early pregnancy in male or female patients, even if treated with them at the time of conception [37,38,39,40,41,42,43]. This is reassuring for doctors who are unsure of the risk they are exposing patients to with their therapy. Analyses of data from safety reports and registries are especially pertinent given that female patients who are pregnant or wishing to conceive are often excluded from clinical trials or their participation during trials is interrupted in the case of unintended pregnancy. Therefore, we have missing data for this patient group and need real-world data (e.g., from registries) to fill this gap in knowledge. Data from pregnancies exposed to biologics should be urgently collected and analysed to gain more evidence for safe treatment options. Collected data from safety registries or real-world data exist for biologics of all target groups, especially for the TNF alpha blockers with first approvals from 2004, which lead to cautious recommendations or phrasing in guidelines [6,7,44,45]. Different medical specialties use biologics for various diseases, e.g., inflammatory bowel diseases or rheumatoid disease [46,47,48], and they are all aiming to provide patients with reduced burden to live their lives as normally as possible. Therefore, we should also exchange interdisciplinary knowledge and data to empower real-world evidence for safe therapy in conception, pregnancy and breast feeding.

### Limitations

As expected, the response rate was around 30%, as this is a sensitive and personal issue for patients. Although the survey was sent with two different questionnaires for each sex, men answered the questions in some cases for pregnancy issues of their partners, and we eliminated these questionnaires with detection. Not all patients filled out all questions and contributed to missing data. Moreover, the questionnaire was self-reported. The answers reflect the patients’ perspective without confirmation by the treating physicians, which may account for potential bias. With this survey conducted, we have no information other than the patients’ view on their disease and treatment; therefore, we cannot make any statement about the course of the psoriasis during pregnancy. We know that this disease can improve in about half of the cases during pregnancy but can also worsen in about a quarter of cases and may even flare up after delivery [49]. The survey was sent by mail without direct contact to the respondents and, consequently, no possibility to ensure proper understanding of the questions.

## 5. Conclusions

The findings of this project emphasize the importance of well-conducted information and communication between patients and physicians regarding family planning and therapy options. Although the wish to conceive in this age group of patients between age 18 and 55 is natural and the disease with PASI 90–100 goals enables normal appearance, social life and family planning, the topic of family planning and conception wishes is often overlooked. Patients do not approach treating physicians proactively with this topic, even if they have family planning in mind. Likewise, when medical specialists do not broach the topic of family planning, this leaves patients to seek information from the internet and other media outlets with potentially low quality. There is a need to lead the discussion about family planning, conception and pregnancy with the patients and support them with evidence-based information to enable the use of all therapy options, both available and compatible with these wishes.

## Figures and Tables

**Table 1:** Sociodemographic and clinical data of survey responders and non-responders stratified by gender. Significant *p*-values (*p* ≤ 0.05) are highlighted bold.

***Men***											
	Responder to survey					Non-Responder to survey					
	N	Min	Max	Mean/%	SD	N	Min	Max	Mean/%	SD	*p*-value
Age	248	18.0	49.0	38.4	7.4	548	18.0	52.0	36.9	8.3	**0.014**
BMI	244	19.8	54.9	28.4	5.3	538	17.2	56.3	28.4	5.4	0.970
PsA (%)	248	n.a	n.a	19.4	n.a	548	n.a	n.a	18.6	n.a	0.804
PASI	241	0.0	59.4	16.4	11.0	533	0.0	57.6	16.3	10.7	0.990
BSA	236	0.0	100.0	25.6	20.1	525	0.0	100.0	26.4	21.2	0.604
DLQI	229	0.0	28.0	10.5	6.8	510	0.0	30.0	12.1	7.6	**0.005**
***Women***											
	Responder to survey					Non-Responder to survey					
	N	Min	Max	Mean/%	SD	N	Min	Max	Mean/%	SD	*p*-value
Age	150	21.0	51.0	37.6	7.7	348	18.0	52.0	35.8	8.4	**0.026**
BMI	147	18.4	52.1	28.3	7.2	345	15.6	57.9	28.1	7.2	0.798
PsA (%)	150	n.a	n.a	22.7	n.a	348	n.a	n.a	21.3	n.a	0.728
PASI	146	0.6	57.6	14.4	9.0	344	0.0	63.6	14.7	10.2	0.747
BSA	142	1.0	90.0	25.4	20.9	332	0.0	90.0	24.3	19.9	0.585
DLQI	145	0.0	29.0	14.3	7.4	316	0.0	30.0	14.1	7.4	0.780

**Table 2:** Current systemic therapy of survey responders (classified).

	Men Surveyed with Wish to Conceive (*n* = 20)	Men Surveyed Without Wish to Conceive (*n* = 229)	*p*	Women Surveyed with Wish to Conceive (*n* = 19)	Women Surveyed Without Wish to Conceive (*n* = 125)	*p*
**Biological treatment**	17 (85.0)	190 (82.9)	>0.999	13 (68.4)	81 (64.8)	>0.999
TNF-alpha inhibitors (other than Certolizumab)	4 (20.0)	28 (12.2)	>0.999	1 (5.3)	6 (4.8)	>0.999
Certolizumab	0 (0.0)	0 (0.0)	n.a	3 (15.8)	1 (0.8)	0.113
IL-17 inhibitors	7 (35.0)	73 (31.9)	>0.999	4 (21.1)	29 (23.2)	>0.999
IL-23 inhibitors or IL-12/23 inhibitors	6 (30.0)	89 (38.8)	>0.999	5 (26.3)	45 (36.0)	>0.999
Other biological treatment	0 (0.0)	0 (0.0)	n.a	0 (0.0)	0 (0.0)	n.a
**Non biological treatment**	2 (10.0)	20 (8.7)	>0.999	1 (5.3)	15 (12.0)	>0.999
PDE-4 inhibitors (Apremilast)	0 (0.0)	2 (0.9)	>0.999	0 (0.0)	0 (0.0)	n.a
Ciclosporin	0 (0.0)	0 (0.0)	n.a	0 (0.0)	0 (0.0)	n.a
Methotrexate (MTX)	2 (10.0)	13 (5.7)	>0.999	0 (0.0)	10 (8.0)	>0.999
Fumaric acid esters	0 (0.0)	5 (2.2)	>0.999	1 (5.3)	3 (2.4)	>0.999
JAK inhibitors	0 (0.0)	0 (0.0)	n.a	0 (0.0)	2 (1.6)	>0.999
Other non-biological treatment	0 (0.0)	0 (0.0)	n.a	0 (0.0)	0 (0.0)	n.a
No regular systemic therapy/unknown	2 (10.0)	23 (10.0)	>0.999	5 (26.3)	31 (24.8)	>0.999

*p* Bonferroni adjusted *p*-value; (1) numbers are presented as N (%); (2) numbers do not sum up, because a combination of different therapies is possible (e.g., Adalimumab and MTX).

**Table 3:** Systemic therapy at the time of conception (classified) compared to inclusion medication in the PsoBest-registry. Lines in bold represent the aggregation of the therapies listed below.

	Men: Systemic Therapy at the Time of Fathering (*n* = 47 Events)	Women: Systemic Therapy at the Time of Pregnancy (*n* = 20 Events)	Male PsoBest Patients Registered in 2018/2019 (*n* = 2730)	Female PsoBest Patients Registered in 2018/2019 (*n* = 1888)
**Biological treatment**	**31 (65.9)**	**8 (40.0)**	**1317 (48.2)**	**849 (44.9)**
TNF-alpha inhibitors (other than Certolizumab)	9 (19.1)	2 (10.0)	235 (8.6)	160 (8.5)
Certolizumab	0 (0.0)	3 (15.0)	12 (0.4)	54 (2.9)
IL-17 inhibitors	12 (25.5)	3 (15.0)	566 (20.7)	341 (18.1)
IL-23 inhibitors or IL-12/23 inhibitors	10 (21.3)	0 (0.0)	504 (18.5)	294 (15.6)
Other biological treatment	0 (0.0)	0 (0.0)	0 (0.0)	0 (0.0)
**Non biological treatment**	**6 (12.8)**	**1 (5.0)**	**1464 (53.6)**	**1083 (57.4)**
PDE-4 inhibitors (Apremilast)	0 (0.0)	0 (0.0)	80 (2.9)	93 (4.9)
Ciclosporin	1 (2.1)	0 (0.0)	29 (1.1)	19 (1.0)
Methotrexate	1 (2.1)	0 (0.0)	543 (19.9)	385 (20.4)
Fumaric acid esters	4 (8.5)	1 (5.0)	765 (28.0)	546 (28.9)
JAK inhibitors	0 (0.0)	0 (0.0)	0 (0.0)	0 (0.0)
Other non-biological treatment	0 (0.0)	0 (0.0)	47 (1.7)	40 (2.1)
No regular systemic therapy/unknown	10 (21.3)	11 (55.0)	0 (0.0)	0 (0.0)

(1) numbers are presented as N (%); (2) numbers do not sum up, because a combination of different therapies is possible (e.g., Adalimumab and MTX).

## Data Availability

The data supporting this study are not publicly available due to restrictions established by the PsoBest Registry and European legislation.

## Supplementary Materials

The following supporting information can be downloaded at: https://www.mdpi.com/article/10.3390/healthcare13091017/s1, Figure S1. Survey form for men. Figure S2. Survey form for women.
