# Supplementary material for: Communication and Therapy Planning for Patients of Reproductive Age Under Immunomodulatory Treatments for Psoriasis or Psoriatic Arthritis—Survey of the German National Psoriasis Registry PsoBest
Source: Healthcare (Basel). 2025 Apr 28;13(9):1017. doi: 10.3390/healthcare13091017 (PMC12071467; doi:10.3390/healthcare13091017)
Supplement: Supplementary file 1 [file healthcare-13-01017-s001.zip › healthcare-3489872-supplementary.pdf]

Supplementary Materials

ID: <xxxxx>

Erhebung zur Arzneimitteltherapie bei Kinderwunsch und Zeugung
Männliche PsoBest-Teilnehmer

1. Besteht bei Ihnen ein Kinderwunsch?

□ Nein (Weiter mit Frage 2) □ Ja □ Ich plane eine Zeugung in absehbarer Zeit.
□ Ich lasse eine Zeugung spontan auf mich zukommen.

2. Erhalten Sie zurzeit eine systemische Therapie (Tablette/Spritze/Infusion) wegen Ihrer Schuppenflechte?

□ Nein □ Ja, folgende: ____

3. Wurde mit Ihnen hinsichtlich der Behandlung Ihrer Schuppenflechte eine Zeugung angesprochen?

□ Nein □ Ja, während der Therapie □ Ja, bereits vor Therapiestart

3.1 <u>Wenn Ja:</u> Durch wen**:** □ Arzt □ Ärztin

Fachrichtung *(z.B. Dermatologie, Rheumatologie):* ____

Was wurde besprochen: ____

4. Fühlen Sie sich von Ihrem Arzt/Ihrer Ärztin zu Ihrer Schuppenflechte und einer Zeugung gut informiert?

□ Gar nicht □ Eher nicht □ Teils, teils □ Eher Ja □ Ja, sehr

5. Haben Sie sich noch weitere Informationen bezüglich Kinderwunsch und Therapie Ihrer Schuppenflechte geholt?

□ Nein □ Ja, wo haben Sie sich informiert: ____

Die nachfolgenden Fragen sollen uns helfen, den Zusammenhang zwischen der Therapie Ihrer Schuppenflechte und einer Zeugung besser zu verstehen. Sollten Sie hierzu Fragen haben oder weitere Informationen wünschen, wenden Sie sich an Ihren behandelnden Arzt/Ihre behandelnde Ärztin.

6. Haben Sie unter Therapie Ihrer Schuppenflechte ein Kind gezeugt?

□ Nein (Weiter mit Frage 7) □ Ja (Weiter mit Frage 6.1) □ Ja, mehrmals (Weiter mit Frage 6.1)

	**1. Zeugung**	**2. Zeugung**	**3. Zeugung**	**Weitere Zeugungen**
**6.1 Welches systemische Medikament (Tablette/Spritze/Infusion) haben Sie zur Zeit der Zeugung erhalten?**	__________	__________	__________	________________

Bitte wenden.

ID: <xxxxx>

	1. Zeugung		2. Zeugung		3. Zeugung		Weitere Zeugungen	
**6.2 Wie war der Ausgang Ihrer Zeugung?**		**Nicht bekannt**		**Nicht bekannt**		**Nicht bekannt**		**Nicht bekannt**
Gesundes Kind geboren in Woche (SSW):	____SSW	O	____SSW	O	____SSW	O	____SSW	O
Frühgeburt in Woche (SSW):	____SSW	O	____SSW	O	____SSW	O	____SSW	O
Kindsgewicht* (g):	_______g	O	_______g	O	_______g	O	_______g	O
Kindsgröße* (cm):	_____cm	O	_____cm	O	_____cm	O	_____cm	O
Geschlecht des Kindes* (m, w, d):		O		O		O		O
Die Schwangerschaft meiner Partnerin wurde vorzeitig beendet in Woche (SSW):	____SSW	O	____SSW	O	____SSW	O	____SSW	O
Warum?	________	O	________	O	________	O	________	O
Meine Partnerin hatte eine Fehlgeburt in Woche (SSW):	____SSW	O	____SSW	O	____SSW	O	____SSW	O
Anderer Ausgang:	________	O	________	O	________	O	________	O

** Bei Mehrlingen können Sie die Angaben durch einen Querstrich trennen, z.B. m/w/w*

7. Haben Sie das Gefühl, dass Ihre Schuppenflechte einen Einfluss auf eine Zeugung hatte?

□ Gar nicht □ Eher nicht □ Teils, teils □ Eher Ja □ Ja, sehr

Wenn ja, welchen vermuten Sie: ____

7.1 Haben Sie darüber mit Ihrem Arzt/Ihrer Ärztin gesprochen?

□ Nein □ Ja

8. Wurde Ihre Therapie bei geäußertem Kinderwunsch verändert oder gewechselt?

□ Nein □ Ja, ein anderes Medikament wurde gewählt.

□ Ja, mein Medikament wurde vor der Zeugung abgesetzt. □ Anderes: ______________________

9. Möchten Sie noch etwas anmerken?

____

Ausfülldatum: ____.____.________ (TT. MM. JJJJ)

Herzlichen Dank für Ihre Teilnahme!

Ihr PsoBest-Team

Bitte nur die 2 Fragebogen-Seiten im beigefügten Rückumschlag oder per Fax (040-7410-44487) zurücksenden, Danke!

Figure S1. Survey form for men. *

* Translated questions as follows (by authors):

- Question 1: Do you wish to conceive? (Yes, No, I am planning to conceive in the foreseeable future, I want to conceive spontaneously).
- Question 2: Are you currently receiving systemic therapy for your psoriasis?
- Question 3: Has family planning been discussed with you regarding the treatment of your psoriasis?
- Question 4: Do you feel well informed by your doctor about your psoriasis in terms of fertility and treatment?
- Question 5: Have you obtained any further information regarding family planning and treatment of your psoriasis? Where did you obtain information?
- Question 6: Have you fathered a child under antipsoriatic treatment? 6.1. systemic medication at the time of conception, 6.2. How was the outcome of the pregnancy? (healthy child, preterm birth, gestational age, weight, height, sex, My partner's pregnancy was terminated prematurely, My partner had a miscarriage.)
- Question 7: Do you feel that your psoriasis had an impact on conception? 7.1. Have you spoken to your doctor about this?
- Question 8: Was your therapy changed or switched when you expressed a wish to conceive? (No, Yes, another medication was chosen. Yes, my medication was discontinued before conception.)
- Question 9: Is there anything else you would like to mention?

ID: <xxxxx>

Erhebung zur Arzneimitteltherapie bei Kinderwunsch und Schwangerschaft
Weibliche PsoBest-Teilnehmerinnen

1. Besteht bei Ihnen ein Kinderwunsch?

□ Nein (Weiter mit Frage 2) □ Ja □ Ich plane eine Schwangerschaft in absehbarer Zeit.
□ Ich lasse eine Schwangerschaft spontan auf mich zukommen.

2. Erhalten Sie zurzeit eine systemische Therapie (Spritze/Tablette/Infusion) wegen Ihrer Schuppenflechte?

□ Nein □ Ja, folgende: ____

3. Wurde mit Ihnen im Rahmen der Behandlung Ihrer Schuppenflechte eine Schwangerschaft angesprochen?

□ Nein □ Ja, während der Therapie □ Ja, bereits vor Therapiestart

3.1 <u>**Wenn Ja:**</u> Durch wen**:** □ Arzt □ Ärztin

Fachrichtung *(z.B. Dermatologie, Rheumatologie):* ____

Was wurde besprochen: ____

4. Fühlen Sie sich von Ihrem Arzt/Ihrer Ärztin zu Ihrer Schuppenflechte und einer Schwangerschaft gut informiert?

□ Gar nicht □ Eher nicht □ Teils, teils □ Eher Ja □ Ja, sehr

5. Haben Sie sich noch weitere Informationen bezüglich Kinderwunsch und Therapie Ihrer Schuppenflechte geholt?

□ Nein □ Ja, wo haben Sie sich informiert: ____

Die nachfolgenden Fragen sollen uns helfen, den Zusammenhang zwischen der Therapie Ihrer Schuppenflechte und einer Schwangerschaft besser zu verstehen. Sollten Sie hierzu Fragen haben oder weitere Informationen wünschen, wenden Sie sich an Ihren behandelnden Arzt/Ihre behandelnde Ärztin.

6. Sind/waren Sie während einer systemischen Behandlung (Tablette/Spritze/Infusion) der Schuppenflechte schwanger?

□ Nein (Weiter mit Frage 7) □ Ja (Weiter mit Frage 6.1) □ Ja, mehrmals (Weiter mit Frage 6.1)

	**1. Schwanger-schaft**	**2. Schwanger-schaft**	**3. Schwanger-schaft**	**Weitere Schwangerschaften**
**6.1 Welches systemische Medikament haben Sie während der Schwangerschaft erhalten?**	__________	__________	__________	__________

**6.2 Wie ist Ihre Schwangerschaft allgemein (nicht ihre Schuppenflechte) verlaufen?**	**1. Schwanger-schaft**	**2. Schwanger-schaft**	**3. Schwanger-schaft**	**Weitere Schwanger-schaften**
Ich hatte <u>keine</u> Schwangerschaftskomplikationen, (unauffällige Schwangerschaft).	O	O	O	O
Ich hatte allgemein <u>Beschwerden</u> in der Schwangerschaft, (kein Krankenhausaufenthalt).	O	O	O	O
Ich hatte Beschwerden in der Schwangerschaft, die stationär <u>im Krankenhaus</u> behandelt wurden.	O	O	O	O
**Folgende Beschwerden sind bei mir aufgetreten:**				
Bluthochdruck	O	O	O	O
Schwangerschaftsdiabetes	O	O	O	O
vorzeitige Wehen	O	O	O	O
Blutungen	O	O	O	O
Schwangerschaftserbrechen	O	O	O	O
Anderes	___________	___________	___________	___________

Bitte wenden.

ID: <xxxxx>

Wie war der Ausgang Ihrer Schwangerschaft?	1. Schwanger-schaft	2. Schwanger-schaft	3. Schwanger-schaft	Weitere Schwanger-schaften
Ich habe spontan entbunden.	O	O	O	O
Die Geburt wurde eingeleitet.	O	O	O	O
Die Entbindung erfolgte natürlich (vaginal).	O	O	O	O
Die Entbindung erfolgte mit Kaiserschnitt.	O	O	O	O
Gesundes Kind geboren in Woche (SSW):	_______SSW	_______SSW	_______SSW	______________SSW
Frühgeburt in Woche (SSW):	_______SSW	_______SSW	_______SSW	______________SSW
Kindsgewicht* (g):	_________g	_________g	_________g	______________g
Kindsgröße* (cm):	_________cm	_________cm	_________cm	______________cm
Geschlecht des Kindes* (m, w, d):				
Die Schwangerschaft wurde vorzeitig beendet in Woche (SSW):	_______SSW	_______SSW	_______SSW	______________SSW
Warum?	___________	___________	___________	________________
Ich hatte eine Fehlgeburt in Woche (SSW):	_______SSW	_______SSW	_______SSW	______________SSW
Anderer Ausgang:	___________	___________	___________	________________

** Bei Mehrlingen können Sie die Angaben durch einen Querstrich trennen, z.B. m/w/w*

6.3 Wie entwickelte sich Ihre Schuppenflechte in der Schwangerschaft?	1. Schwanger-schaft	2. Schwanger-schaft	3. Schwanger-schaft	Weitere Schwanger-schaften
In folgenden Dritteln der Schwangerschaft:	1. /2. /3. Drittel	1. /2. /3. Drittel	1. /2. /3. Drittel	1. /2. /3. Drittel
hatte ich eine Verbesserung der Schuppenflechte	O O O	O O O	O O O	O O O
war die Schuppenflechte unverändert	O O O	O O O	O O O	O O O
verschlechterte sich die Schuppenflechte	O O O	O O O	O O O	O O O
Nach der Entbindung:				
hatte ich eine Verbesserung der Schuppenflechte	O	O	O	O
war die Schuppenflechte unverändert	O	O	O	O
verschlechterte sich die Schuppenflechte	O	O	O	O

7. Haben Sie das Gefühl, dass Ihre Schuppenflechte einen Einfluss auf eine Schwangerschaft hatte?

□ Gar nicht □ Eher nicht □ Teils, teils □ Eher Ja □ Ja, sehr

Wenn ja, welchen vermuten Sie: ____

7.1 Haben Sie darüber mit Ihrem Arzt/Ihrer Ärztin gesprochen?

□ Nein □ Ja

8. Wurde Ihre Therapie bei geäußertem Kinderwunsch verändert oder gewechselt?

□ Nein □ Ja, ein anderes Medikament wurde gewählt.

□ Ja, mein Medikament wurde vor der Schwangerschaft abgesetzt. □ Anderes: ________________

9. Möchten Sie noch etwas anmerken?

____

Ausfülldatum: ____.____.________ (TT. MM. JJJJ)

Herzlichen Dank für Ihre Teilnahme!

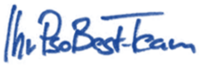

Bitte nur die 2 Fragebogen-Seiten im beigefügten Rückumschlag oder per Fax (040-7410-44487) zurücksenden, Danke!

Figure S2. Survey form for women. *

Translated questions as follows (by authors):

- Question 1: Do you wish to conceive? (Yes, No, I am planning to conceive in the foreseeable future, I want to conceive spontaneously).
- Question 2: Are you currently receiving systemic therapy for your psoriasis?
- Question 3: Has family planning been discussed with you regarding the treatment of your psoriasis? (No, Yes during therapy conduct, yes before initiation of therapy)
- Question 4: Do you feel well informed by your doctor about your psoriasis in terms of fertility and treatment?
- Question 5: Have you obtained any further information regarding family planning and treatment of your psoriasis? Where did you obtain information?
- Question 6: Have you been pregnant under antipsoriatic treatment? 6.1. systemic medication at the time of conception, 6.2. How was Your pregnancy? (no complications, general complaints with/without admission to hospital, special comorbidities of interest like hypertension, gestational diabetes, preterm labour, bleeding, hyperemesis), 6.3. How was the outcome of the pregnancy? (spontaneous delivery, induced delivery, vaginal delivery, caesarean section, healthy child, preterm birth, gestational age, weight, height, sex, premature termination, miscarriage.), 6.3. How was the clinical appearance of Your psoriasis during pregnancy in which trimester? (improvement, unchanged, worsening) How was Your psoriasis after delivery? (improvement, unchanged, worsening)
- Question 7: Do you feel that your psoriasis had an impact on your pregnancy? 7.1. Have you spoken to your doctor about this?
- Question 8: Was your therapy changed or switched when you expressed a wish to conceive? (No, Yes, another medication was chosen. Yes, my medication was discontinued before conception.)
- Question 9: Is there anything else you would like to mention?
